# Recent Advance in Marine Polysaccharides: Structure, Anti-Inflammatory Mechanisms, and Functional Applications

**DOI:** 10.3390/md24040129

**Published:** 2026-03-31

**Authors:** Yuchen Wang, Jingyi Luo, Chao Xu, Dongyu Hu, Yimeng Li, Yanzuo Ye, Jun Yang, Xianxiang Chen, Chuan Li, Kexue Zhu

**Affiliations:** 1 School of Food Science and Engineering, Hainan University, Haikou 570228, China; 20243003978@hainanu.edu.cn (Y.W.); 20233001093@hainanu.edu.cn (J.L.);; 2 Department of Clinical Nutrition, Haikou Affiliated Hospital of Central South University Xiangya School of Medicine, Haikou 570208, China; 3 Spice and Beverage Research Institute, Chinese Academy of Tropical Agricultural Sciences, Wanning 571533, China

**Keywords:** marine polysaccharides, structural characteristics, anti-inflammatory mechanisms, functional applications

## Abstract

Inflammation is pivotal to the pathogenesis of chronic disorders, including diabetes and cardiovascular disorders. Conventional pharmaceuticals used in the treatment of inflammation and related diseases face several challenges. In recent years, polysaccharides isolated from marine organisms have attracted extensive research attention due to their good safety profile, easy availability, and powerful anti-inflammatory properties. However, there is still a lack of systematic elucidation of their anti-inflammatory mechanisms and functional effects. In this review, the sources and structural characteristics of marine polysaccharides were reviewed. Moreover, the anti-inflammatory mechanisms of marine polysaccharides and their advanced applications were discussed. Finally, the current challenges of marine polysaccharides in anti-inflammatory research and food industry applications, as well as future research directions, were proposed. This review deepens the understanding of the anti-inflammatory effects of marine polysaccharides and provides feasible guidance for the development and clinical application of novel anti-inflammatory drugs.

## 1. Introduction

Inflammation is a fundamental and important protective mechanism in the human body. Essentially, it is an immune defense response of organisms to harmful factors such as physical damage, pathogen invasion and chemical stimulation. The inflammatory response removes the above harmful stimuli and repairs tissues by releasing inflammatory mediators such as interleukin (IL)-1, tumor necrosis factor (TNF)-α, activated cyclooxygenase (COX-2) and inducible nitric oxide synthase (iNOS) [1]. However, chronic diseases such as atherosclerosis, rheumatoid arthritis and inflammatory bowel disease may occur and develop when chronic inflammation lasts for a long time and is uncontrolled. Inflammation may also increase the risk of cancer [2]. Currently, nonsteroidal anti-inflammatory drugs (NSAIDs) and corticosteroids represent the main pharmacological options for controlling inflammation in clinical settings. Although these drugs can quickly relieve inflammatory symptoms, NSAIDs easily cause gastric mucosal damage, ulcers and other gastrointestinal adverse reactions, and steroids may cause osteoporosis, immunosuppression and other problems. Long-term use may cause significant disadvantages [3]. Therefore, there is an urgent need to explore alternative treatment strategies that are safe, green, and low-toxicity.

Marine polysaccharides are natural biological macromolecules, which are mainly extracted from algae, marine animals and microorganisms. These types of macromolecules form linear or branched structural features by connecting repeating monosaccharide units through glycosidic bonds [4]. The sulfate and hydroxyl groups they contain give marine polysaccharides and their derivatives good water solubility and high viscosity. The sulfate groups can also enhance their binding ability with proteins, lipids, and other biomolecules [5]. They achieve mild and multi-target anti-inflammatory intervention effects by regulating key signaling pathways such as NF-κB and MAPK [6]. The advantages of marine polysaccharides compared with traditional drugs are that they have low toxicity and are suitable for long-term clinical treatment. At the same time, these natural biomolecules possess diverse biological activities, particularly in antioxidant defense and immune regulation [7]. Moreover, by combining with a variety of substances, they can achieve precise targeted delivery to the site of inflammation. Because they are less likely to induce drug resistance and are rich in raw material resources, marine polysaccharides have shown great potential in the development of functional foods and anti-inflammatory drugs.

The structure of a substance generally determines its properties, and the same is true for marine polysaccharides. Molecular weight, glycosidic residue composition, glycosidic bond type, and degree of sulfation are structural factors that directly determine the intensity of anti-inflammatory activity, target selection, and mechanism specificity of marine polysaccharides [8]. However, most marine polysaccharides have extremely complex and multi-target anti-inflammatory mechanisms. For example, they can protect the structural integrity of cells by scavenging free radicals. They can also reduce advanced oxidation protein products (AOPPs) and enhance antioxidant activity. In addition, they can regulate not only immune cell activity but also inflammatory factor levels to achieve anti-inflammatory effects [9]. Therefore, to deeply explore the anti-inflammatory potential of these polysaccharides, the primary prerequisite is to clarify their structural basis and establish reliable structure–activity relationships. This is also one of the core focuses of this review.

This review aims to comprehensively summarize the anti-inflammatory potential of marine polysaccharides as a natural alternative to traditional anti-inflammatory drugs. Specifically, this review first comprehensively summarizes the diversity characteristics of marine polysaccharides from the perspectives of source and structure. Furthermore, this review elaborates on the anti-inflammatory molecular mechanisms of marine polysaccharides and their applications in related fields. Finally, the development prospects and practical challenges of the transformation of anti-inflammatory properties of marine polysaccharides are analyzed, aiming to provide theoretical support for the research and development of anti-inflammatory drugs and assist in their large-scale application and industrial transformation.

Methodology: This review was conducted through an extensive and in-depth literature search of peer-reviewed publications spanning from 2013 to 2026, utilizing the PubMed, Google Scholar, and Scopus databases. The search strategy employed key terms such as “marine polysaccharides,” “anti-inflammatory activity,” “structure-activity relationship,” and “clinical application.” Articles were selected based on their direct relevance to the anti-inflammatory properties of marine polysaccharides with original research studies and authoritative review papers. In total, 124 publications were included in this analysis, which collectively provide a comprehensive overview of both foundational knowledge and recent advancements in the field.

## 2. Structure–Activity Relationship of Marine Polysaccharides

**Figure 1:**
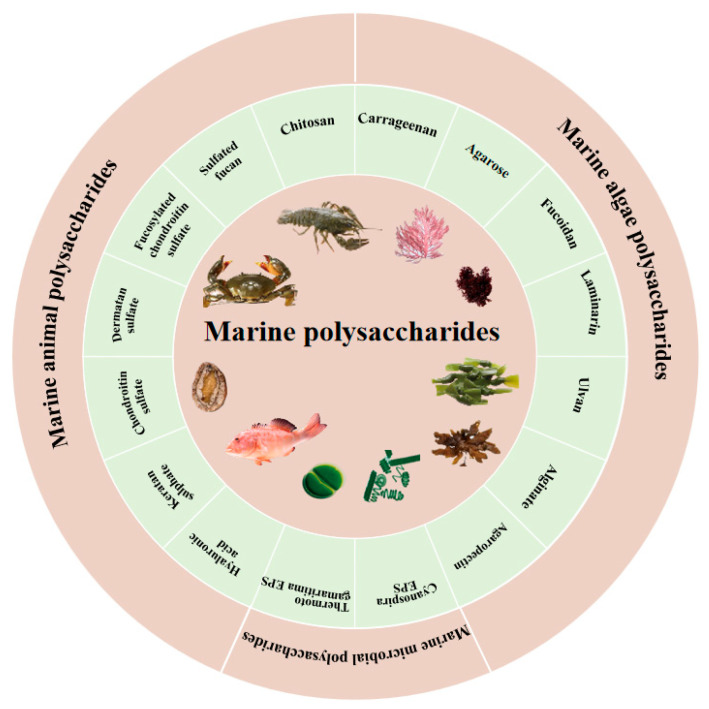
Main types of marine polysaccharides.

Marine polysaccharides exhibit different structures in the composition of sugar chains, linkage modes, substituent groups, and molecular weight distribution, which influence their anti-inflammatory effects [10]. This structural information is a crucial basis for researching functional properties and developing novel food formulations. Hence, it is necessary to elucidate the structural characteristics of marine polysaccharides in terms of their main types and elaborate on their structural diversity and molecular features with the aid of commonly used molecular characterization approaches. The main types of marine polysaccharides are shown in Figure 1.

### 2.1. Structural Characteristics of Marine Polysaccharides

The unique conditions of the marine environment, such as high salinity, high hydrostatic pressure, low temperature, and weak light, have prompted marine organisms to evolve polysaccharides with diverse structures and specific functions [11]. Notably, marine polysaccharides with significantly anti-inflammatory activities are primarily derived from algae, animals, and microorganisms. Most belong to sulfated polysaccharides, which have attracted extensive research because of their unique chemical structure and strong bioactivity. Sulfated polysaccharides are high-value active components in marine polysaccharides. The introduction of sulfate groups not only changes the original physical and chemical properties of polysaccharides but also endows them with significant antioxidant, antitumor, and immunomodulatory activities [12] (summarized in Table 1).

#### 2.1.1. Algal Polysaccharides

Algae is one of the main sources of marine polysaccharides. Owing to the different metabolic pathways of different species of algae, these polysaccharides from algae show significant structural diversity and usually have unique functional group modifications [22].

Brown algae polysaccharides mainly include alginate [23], fucoidan [24], and laminarin. Alginate is a type of linear polymer, which is mainly formed by β-D-mannuronic acid (M) and α-L-guluronic acid (G) via 1,4-glycosidic bonds [13]. Furthermore, the physicochemical and biological properties, such as gelation, viscosity, and bioactivity, are decided based on the ratio of M and G, the sequence arrangement (such as MM segments, GG segments, and MG segments), and pH conditions [25]. Fucoidan is formed by connecting L-fucose as the main structural unit through 1,3- or 1,4-glycosidic bonds. The molecule also contains trace amounts of mannose, galactose, and glucuronic acid residues; the sulfate groups are mainly directionally substituted at the C2 and C4 positions of fucose. In addition, the structures are still modified with acetyl groups, which are the key factors influencing their bioactivities, such as anti-inflammatory and anticoagulant effects [14]. Laminarin is a water-soluble polysaccharide extracted from kelp and wakame. As a type of β-1,3-glucan, it consists of D-glucose units connected by 1,3- and 1,6-glycosidic linkages, which exhibit great anti-inflammatory effects [15].

Red algal polysaccharides mainly include carrageenan [26] and agar [27]. Carrageenan is a linear sulfated polysaccharide formed by the alternating linkage of β-D-galactopyranose (namely G-units) and α-D-galactopyranose (namely D-units) through 1,3- and 1,4-glycosidic bonds, or by 3,6-anhydro-α-D-galactopyranose (namely DA-units) [14,16]. According to the difference in substitution sites of the sulfate group, carrageenan can be divided into three main types. The degree of substitution of κ-carrageenan is 1, and only the sulfate group is bound at the C4 site of β-D-galactose (G4S-DA) in each disaccharide unit, which means κ-carrageenan exhibits excellent gelling ability and forms strong, rigid, brittle gels in the presence of K^+^, and its gel strength is the highest among the three types [16,28]. The substitution degree of ι-carrageenan is 2, and the substitution sites are C4 and C2 of β-D-galactose and 3,6-anydro-α-D-galactose (G4S-DA2S), respectively, which means that it shows moderate gelling ability and forms soft, elastic gels with medium gel strength in the presence of Ca^2+^ [16,28]. The degree of substitution of λ-carrageenan is 3, which is the highest among the three kinds of carrageenan. It is linked at the C2 position of β-D-galactose and at C2 and C6 positions of α-D-galactose (G2S-D2S,6S), which means λ-carrageenan does not gel and only functions as a thickener to increase viscosity without forming a gel network [16,17,26]. Agar includes agarose and agaropectin. The former is a linear polysaccharide without sulfate group substitution. It brings β-D-galactose and 3,6-dehydrated -α-L-galactose as structural units and forms molecular chains through 1,3- and 1,4-glycosidic bonds. It has both excellent gel properties and good biocompatibility. Agaropectin, due to containing a small amount of sulfate groups and carboxyl groups, has a significant impact on the physical and chemical properties of agar [11].

Green algal polysaccharides mainly include ulvan [29]. Ulvan is a sulfated polysaccharide amply present in the cell walls of green seaweeds of the genus *Ulva* [18]. It is mainly a polyanionic heteropolysaccharide composed of rhamnose, glucuronic acid, iduronic acid, and xylose and contains sulfate groups [17]. Its skeleton is mainly composed of α- and β-(1,4)-bonds, forming repeated disaccharide units. The two main repeating disaccharide units are aldobiuronic acids, known as ulvanobiuronic acid 3-sulfate type A (A_3s_), and type B (B_3s_), and in some cases there are minor disaccharide units known as type-U ulvanobioses (U_3s_ and U_2′s,3s_) [18]. The main structure of A3s is β-D-glucuronic acid (1 → 4)-α-L-rhamnose-3-sulfate. The structure of B3s is α-L-iduronic acid (1 → 4)-α-L-rhamnose-3-sulfate. In addition, the U_3s_ type is composed of xylose instead of uronic acid [β-D-xylose (1 → 4)-α-L-rhamnose-3-sulfate], while in the U_2′s,3s_ type, the uronic acid is replaced by sulfated xylose [β-D-xylose 2-sulfate (1 → 4)-α-L-rhamnose-3-sulfate]. The main chain is linear, and there are some sulfated O-2 sites and a small number of branched chains (such as xylose/glucuronic acid side chains) [18,30,31]. In a word, the monosaccharide composition of green algae polysaccharides is relatively complex, with common components including galactose, arabinose, and xylose, among others. Meanwhile, their sulfation modification patterns also exhibit diversity. Their anti-inflammatory activity is often positively correlated with the content of sulfate groups, and this characteristic also confirms the importance of this structure [32].

#### 2.1.2. Animal Polysaccharides

Marine animals are also an important source of marine polysaccharides. Chondroitin sulfate and dermatan sulfate can be derived from the cartilage and skin of marine animals. Chitosan can originate from the shells of marine animals.

Chondroitin sulfate is primarily extracted from the cartilage tissues of cartilaginous fish such as sharks and rays. It is a linear polysaccharide whose main chain consists of two monomers, D-glucuronic acid and N-acetyl-D-galactosamine, linked through β-1,3- and β-1,4-glycosidic bonds. Most of the sulfate groups on its structure are attached to the C4 or C6 position of N-acetyl-D-galactosamine residues [12]. Based on the differences in the substitution positions of sulfate groups, chondroitin sulfate can be divided into multiple subtypes: the sulfate group of chondroitin sulfate A is attached to the C4 position and the sulfate group of chondroitin sulfate C is located at the C6 position, while chondroitin sulfate D undergoes sulfation modification at both the C2 position of glucuronic acid and the C6 position of galactosamine. Compared with monosulfated chondroitin sulfate, disulfated polysaccharides of this type exhibit stronger anti-inflammatory potential [4]. The anti-inflammatory effect of chondroitin sulfate is mainly achieved by inhibiting the release of inflammatory factors and protecting chondrocytes [33].

Dermatan sulfate is widely distributed in the skin, blood vessel walls, and cartilage tissues of marine mammals, and its molecular structure is relatively similar to that of chondroitin sulfate. Its main chain is composed of L-iduronic acid (or D-glucuronic acid) and N-acetyl-D-galactosamine connected via β-1,3- and β-1,4-glycosidic linkages [19]. Sulfate groups are located at the C4 position of N-acetyl-D-galactosamine, while partial sulfation also occurs at the C2 position of iduronic acid. The presence of these sulfate groups leads to better biological activity and the possession of various physiological functions [34].

Chitosan stands out as a distinctive cationic polysaccharide. It is prepared through a deacetylation reaction of chitin from crustacean shells, insect exoskeletons and fungal cell walls, and it is the only natural alkaline polysaccharide discovered to date [35]. Chitosan is a linear polysaccharide with a core (1,4)-2-amino-2-deoxy-β-D-glucan molecular structure. The structural and functional properties of chitosan are mainly governed by its degree of deacetylation (DD) and molecular weight (Mw) [20]. The DD refers to the proportion of amino groups (-NH_2_) in chitosan molecules, typically ranging from 50% to 100%. Deacetylation critically shapes a range of key chitosan properties including acid-base behavior, biodegradability, and metal ion chelating, thereby dictating its classification and end-use applicability [36]. Besides the degree of deacetylation, a key parameter, molecular weight, is also an important factor affecting the physicochemical characteristics and bioactivities of chitosan [20]. The solubility of high-molecular-weight chitosan in water is lower than that of low-molecular-weight chitosan, and low-molecular-weight chitosan has better low-temperature protection [35]. Existing studies have confirmed that low-molecular-weight chitosan can penetrate bacterial cell walls and inhibit the mRNA synthesis and DNA transcription of bacteria and that its anti-inflammatory effect is better than that of high-molecular-weight chitosan [37].

#### 2.1.3. Microbial Polysaccharides

Marine microbial polysaccharides are a class of biological macromolecules synthesized by marine microorganisms such as bacteria and fungi during their metabolic activities, and their structures exhibit extremely rich diversity characteristics [38]. The constituent units of this type of polysaccharide include monosaccharide residues such as glucose, galactose, mannose, and rhamnose, and these residues form stable linkage structures through different types of glycosidic bonds. Furthermore, the structure of the polysaccharides is altered due to their production by different bacterial strains, their substitution sites and their contents of sulfate groups. *Vibrio* sp. KMM 8419 is a *Gram-negative bacterium* that was isolated from the mucus of the marine polychaete *Chaetopterus cautus* collected from the Sea of Japan. The structure of its sulfated capsular polysaccharide is highly unusual and exhibits remarkable structural complexity and diversity. The sulfate group is attached to C-3 of the L-rhamnose residue, while acetylation modification occurs at C-4, which affects the overall charge distribution, water solubility and binding interactions with other biological molecules. The degree of sulfation and the specificity of modification sites play a key role in maintaining bacterial biological functions, interactions, cell recognition and other processes [21].

### 2.2. Structural–Functional Relationship of Marine Polysaccharides

Structural analysis is a key step in studying the structure–functional relationship of marine polysaccharides. It mainly focuses on the primary structure of polysaccharides, with emphasis on analyzing molecular weight, monosaccharide composition, and types of glycosidic bonds, as well as the types, contents, and substitution sites of non-sugar groups [39]. In the analysis process, purity testing is the primary step, and key indicators including total sugar and uronic acid content need to be measured with emphasis [2]. (Structural–functional relationships of marine polysaccharides are summarized in Table 2).

#### 2.2.1. Molecular Weight

Molecular weight is a key physical indicator of marine polysaccharides, as it directly affects marine polysaccharides’ solubility, viscosity, biological activity, and anti-inflammatory properties, among others. Due to differences in sources, structural composition, and modifying groups, different types of marine polysaccharides exhibit significant variations in their molecular weight characteristics [40]. The molecular weight distribution of alginate is jointly determined by the M/G ratio, monosaccharide sequence structure, and extraction process. There is a commercially available sodium alginate with a molecular weight ranging from 3.2 × 10^4^ to 4 × 10^5^ g/mol, which has a relatively large span. High-molecular-weight alginate with a longer sugar chain structure has stronger gel properties and viscosity, while low-molecular-weight fragments have better solubility and their biological activity is more easily exerted [23]. The molecular weight range of laminarin is relatively large. Studies have revealed a new type of long-chain laminarin (with a molecular weight of approximately 5.79 × 10^4^ g/mol), which has a densely cross-linked network structure with high branching and a helical conformation. It can exert both immune-enhancing and anti-inflammatory activities [41]. Chitosan can be divided into three categories according to molecular weight standards: <50 kDa is low-molecular-weight, 50–150 kDa is medium-molecular-weight, and >150 kDa is high-molecular-weight. High-molecular-weight chitosan has poor solubility but excellent film-forming and flocculating properties due to its longer molecular chains and strong intermolecular hydrogen bonding. In addition, low-molecular-weight chitosan has short molecular chains and small steric hindrance, resulting in significantly improved water solubility and easier penetration through bacterial cell walls [42].

#### 2.2.2. Monosaccharide Composition

Monosaccharide composition forms the foundation of the primary structure in marine polysaccharides. The types and proportions determine the basic molecular framework of polysaccharides and indirectly regulate their biological activities by influencing the charge distribution and spatial conformation of polysaccharides and their interactions with biomolecules. Fucoidan has L-fucose as its main constituent unit, forming the core skeleton of the polysaccharide main chain; at the same time, the molecule also contains small amounts of miscellaneous sugar components, which are important components of its structural characteristics. And the presence of heterosaccharide units can increase the interaction sites between the polysaccharide and inflammatory factors by disrupting the regularity of the main chain, thereby enhancing the anti-inflammatory effect [14]. The differences between different subtypes of carrageenan (κ-type, ι-type, and λ-type) are affected by the number and position of sulfate groups and the content of 3,6-dehydrated galactose [17,26]. This “double galactose + precise sulfation” monosaccharide composition allows the charge density of carrageenan to be precisely regulated through the number of sulfate groups. The disaccharide unit of λ-carrageenan contains three sulfate groups, which is the structural basis for its strongest intramolecular electrostatic repulsion. Therefore, it has the best water solubility among all types. It can more easily bind to positively charged inflammatory factors or proteins through strong electrostatic interactions, providing a basis for its anti-inflammatory applications [26]. The monosaccharide composition of sulfated polysaccharides isolated from green algae is rather complex, consisting of various neutral monosaccharides such as galactose, arabinose, xylose, and glucose, with no single sugar component being absolutely dominant. Sulfate groups are mainly substituted at the C2/C4 positions of galactose or the C3 position of arabinose. Within a certain range, the higher the content of sulfate groups, the stronger the anti-inflammatory activity of green algae polysaccharides usually is [32].

#### 2.2.3. Glycosidic Bond Linkage and Substituent

The connection mode of glycosidic bonds directly determines the sequence and binding characteristics of monosaccharides, which is crucial for the construction of the skeleton of marine polysaccharides. For example, agarose is not modified by substituents. This linear connection without sulfate groups makes it easy to form a double-helix structure between agarose molecules, which means that agarose shows excellent gel properties and biocompatibility [43]. Meanwhile, agaropectin can undergo sulfation modification at the C6 position of β-D-galactose, and the molecule also contains a small number of carboxyl groups. Sulfate groups can interfere with the formation of the double-helix structure, reducing the gel strength. Carboxyl groups can also enhance the adhesion ability between agaropectin cells [11]. Ulvan is a typical sulfated polysaccharide in green algae, reflecting the correlation between glycosidic bond connections, substituent modifications, and the biological functions of marine polysaccharides. Its three types, A-type (A_3s_), B-type (B_3s_), and, in some cases, U-type (U_3s_ and U_2,3s_), reflect different glycosidic bond connection modes. At the same time, the sulfation substitution of ulvan creates a stable negative charge distribution in the molecular chain and forms a unique spatial structure that binds easily to inflammatory factors and immune cell surface receptors. The synergistic effect of specific glycosidic bond connection patterns and fixed sulfation substitution points endows ulvan with a strong ability to recognize and bind to inflammatory mediators and immune cells, giving it significant anti-inflammatory activity [31].

#### 2.2.4. Characteristics of Advanced Structures

The high-level structure of marine polysaccharides mainly shows three-dimensional conformation in aqueous solution, chain flexibility, aggregation and surface morphology, which directly determine the solution properties and anti-inflammatory activity of marine polysaccharides [44]. For instance, chondroitin sulfate generally exists as a flexible random coil in aqueous solution, and its dynamic conformational changes can be precisely regulated by the substitution sites of sulfate groups [12]. The difference in spatial conformation between chondroitin sulfate A and chondroitin sulfate C is mainly determined by the substitution position of the sulfate groups. Sulfation at the C4 position causes the sugar-ring side chain to be closer to the main chain, making the molecular chain relatively compact, while sulfation at the C6 position places the sulfate group at the end of the sugar ring, resulting in a more extended molecular chain. Due to its higher density of sulfate groups, chondroitin sulfate D has stronger intramolecular electrostatic repulsion, has a more loose random coil structure, and can easily penetrate the cartilage matrix and directly act on chondrocytes [45].

## 3. Anti-Inflammatory Mechanisms

The anti-inflammatory effects of marine polysaccharides exert synergistic actions across multiple dimensions and targets by virtue of their unique molecular structural characteristics including the regulation of inflammation-related signaling pathways, modulation of immune cell functional states, and scavenging of harmful products generated during oxidative stress [46].

### 3.1. Intervening in Key Inflammatory Pathways

**Figure 2:**
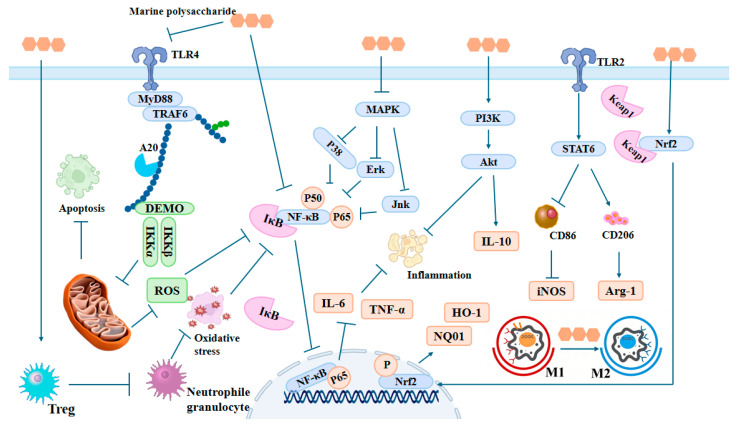
Main pathways of marine polysaccharides for intervening in inflammation.

Marine polysaccharides can inhibit the initiation and amplification of inflammatory cascade reactions from the source by targeting and regulating multiple core inflammatory signaling pathways [5]. The main pathways of marine polysaccharides for intervening in inflammation are shown in Figure 2.

The NF-κB signaling pathway serves as a central regulator in the inflammatory response. Under normal physiological conditions, active NF-κB binds to the inhibitory protein IκB to form a complex, which remains inactive in the cytoplasm [47]. During the inflammatory response, the mechanism by which stimuli like lipopolysaccharide (LPS) activate IKK and phosphorylate and degrade IκB, thereby activating NF-κB to enhance the transcription of pro-inflammatory factors including TNF-α and IL-6, has been extensively investigated and clarified [48]. Sulfated marine polysaccharides can inhibit the activation of the NF-κB pathway. The sulfate groups of polysaccharides bind to IKK, directly blocking its kinase activity and reducing I-κB phosphorylation [49]. The sulfate groups of fucoidan have been demonstrated to ameliorate insulin resistance and attenuate inflammation in obese mice via regulating the PI3K/PKB/GSK-3β pathway and inhibiting JNK as well as IKKβ/NF-κB signaling cascades [50].

The three key branches of the MAPK pathway are ERK, JNK, and p38 MAPK, which play dominant roles in cell proliferation, stress-induced apoptosis, and inflammatory responses, respectively. After activation, they can regulate the transcription process of inflammatory factors through phosphorylation cascades [51]. Signal transduction investigations have revealed that chitosan oligosaccharides can inhibit the phosphorylation of MAPK pathway proteins such as p38, ERK1/2, and JNK and regulate the PI3K/Akt signaling pathway [52]. Low-molecular-weight carrageenan can specifically bind to cell surface receptors through its own sulfate groups, thereby blocking the receptor-mediated phosphorylation process of ERK and p38 MAPK signaling molecules [53]. In addition, because λ-carrageenan contains three sulfate groups per disaccharide unit, its content of sulfate groups is the highest, and its inhibitory effect on the TLR4-mediated MAPK pathway is more obvious than κ-carrageenan and ι-carrageenan [54].

Marine polysaccharides can also regulate the PI3K/Akt pathway to indirectly participate in inflammation inhibition. Chondroitin sulfate can relieve inflammation through activating the PI3K/Akt pathway and upregulating the expression of the anti-inflammatory cytokine IL-10 [55], while inhibiting the release of the pro-inflammatory cytokine IL-1β. This regulatory pattern of balancing “pro-inflammatory and anti-inflammatory” factors can protect chondrocytes and delay the progression of inflammation in chronic inflammatory diseases [56].

### 3.2. Regulating Immune Cells

Immune cells play an important role in the occurrence of inflammatory responses [57]. Marine polysaccharides can reconstruct the immune microenvironment by regulating the activation, polarization direction, and functional phenotype of immune cells, thereby exerting anti-inflammatory effects [58].

The balance of macrophage M1/M2 polarization directly determines the outcome of inflammation regulation. M1 macrophages mainly secrete pro-inflammatory cytokines such as TNF-α and IL-1β, thus promoting inflammatory progression; M2 macrophages release anti-inflammatory mediators such as IL-10 and TGF-β, facilitating the resolution of inflammation [59]. Marine polysaccharides are able to drive macrophage polarization toward the M2 phenotype through multiple mechanisms. Long-chain polysaccharides derived from *Laminaria japonica* (LJPS) can reduce the levels of M1 markers (such as iNOS and CD86) by combining with the Dectin-1 receptor, activating the downstream Syk signaling pathway and upregulating the expression of M2 markers (such as CD206 and Arg-1). Note: Although Dectin-1 is critically involved in immune regulation, it is best known for its role in antifungal immunity and can promote either pro-inflammatory or anti-inflammatory responses in a context-dependent manner. The above pathway therefore represents a plausible mechanism rather than a definitive conclusion. This anti-inflammatory effect might be attributed to Dectin-1’s highly branched helical conformation, which can enhance the binding affinity with receptors and improve the polarization efficiency [41]. Sulfated polysaccharides from green algae (containing mixed sugar units such as galactose and arabinose) promoting M2 polarization exhibited an anti-inflammatory effect by regulating the activation of transcription factor STAT6 and the TLR2 receptor. They also found that the higher the content of sulfate groups, the more significant the polarization effect [60].

Neutrophils are key immune cells recruited in the early stage of inflammation. Their excessive activation releases substances such as neutrophil elastase (NE) and ROS, exacerbating tissue damage [61]. Sulfated polysaccharides, such as dermatan sulfate derived from marine mammals, exert their anti-inflammatory effects through multi-target signal regulation. Due to the special structure of dermatan sulfate with C4 sulfation, it could act as a high-affinity ligand for L-selectin, effectively inhibiting the adhesion and migration of neutrophils, thereby reducing cellular infiltration in inflammatory sites. Its sulfate groups can also directly inhibit the activity of neutrophil elastase (NE) and scavenge ROS, thus alleviating inflammation-mediated tissue damage in the terminal stage [34]. Marine polysaccharides can also interfere with T lymphocyte subsets to exert immunomodulatory effects. Low-molecular-weight chitosan can reduce inflammatory responses by promoting the proliferation of regulatory T cells (Treg cells) and enhancing their immunosuppressive function [62]. Treg cells achieve the alleviating effect on autoimmune inflammation by releasing anti-inflammatory factors such as IL-10 and TGF-β and downregulating the pro-inflammatory activity of Th1 and Th17 cells [63].

### 3.3. Regulating Oxidative Stress and Inflammatory Factors

Oxidative stress and inflammatory responses usually promote each other, exacerbating the reactions and leading to a vicious cycle [64]. Excessive production of ROS activates inflammatory pathways, and the inflammatory response further exacerbates oxidative stress [65]. Marine polysaccharides can exert anti-inflammatory effects by scavenging ROS, regulating the body’s antioxidant system, and directly inhibiting the production and secretion of inflammatory factors, thereby blocking the vicious cycle between oxidative stress and inflammation [66].

Marine polysaccharides’ molecular structures often contain functional groups with antioxidant activity such as hydroxyl groups and sulfate groups [67], so they can act as hydrogen donors or electron donors to directly scavenge and neutralize various reactive oxygen species including superoxide anions (O_2_^−^) and hydroxyl radicals (·OH). For instance, the sulfate groups in fucoidan can directly scavenge free radicals, chelate metal ions, and activate the endogenous antioxidant system, thereby effectively suppressing the vicious cycle between oxidative stress and inflammation [68]. In addition, marine polysaccharides can also regulate the intracellular antioxidant system and exert anti-inflammatory effects through activating the Nrf2 signaling pathway. Low-molecular-weight chitooligosaccharides can reduce Cu^2+^-induced oxidative damage and apoptosis in Nrf2 activation, thereby alleviating oxidative damage [68,69].

In terms of regulating inflammatory mediators, marine polysaccharides can interfere with the generation of inflammatory factors through transcription, translation, and secretion. At the transcriptional level, marine polysaccharides can inhibit NF-κB and MAPK pathways to reduce the gene transcription of pro-inflammatory cytokines such as TNF-α, IL-6, and IL-1β. Studies have indicated that fucoidan can notably reduce the mRNA expression levels of pro-inflammatory cytokines such as TNF-α and IL-6 in obese mouse models by regulating inflammatory pathways and suppressing the activation of JNK and IKKβ/NF-κB signals, thereby improving the inflammatory response associated with insulin resistance [50].

It is worth noting that the regulatory effect of marine polysaccharides on oxidative stress and inflammatory cytokines has structural specificity. The content of sulfate groups is positively correlated with antioxidant and anti-inflammatory activities (for example, λ-carrageenan is better than κ-carrageenan). Low-molecular-weight polysaccharides (such as chitosan < 50 kDa) have a more significant regulatory effect on intracellular ROS scavenging and inflammatory factor transcription than those of high molecular weight due to their strong cell penetration, while polysaccharides with a helical conformation (such as those from *L. japonica* [41]) perform better in activating the antioxidant enzyme system because of their strong binding ability to cell surface receptors.

## 4. Potential Emerging Functional Applications in Food and Biomedical Fields

Marine polysaccharides and their derivatives have excellent anti-inflammatory, antioxidant, biocompatibility and biodegradability and low toxicity. These properties make them ideal candidate materials for advanced applications in food science and biomedical fields. Based on the anti-inflammatory molecular mechanisms described above, these marine-derived polysaccharides have been gradually developed and applied in multiple directions. They are widely used as functional food additives to improve product stability and impart health-promoting functions to foods. At the same time, they serve as potential anti-inflammatory drug candidates for treating intestinal inflammation, osteoarthritis, and oral infections. In addition, they are ideal raw materials for preparing hydrogels for wound repair and tissue regeneration, and can also be used to construct targeted drug delivery systems to improve therapeutic effects and reduce side effects. The anti-inflammatory activity and application status of representative marine polysaccharides are summarized in Table 3, to provide a clear reference for their further research and industrial transformation.

### 4.1. Functional Food Additives

Owing to anti-inflammatory properties and excellent biocompatibility, marine polysaccharides have been widely utilized in the field of food additives. Fucoidan and other algal polysaccharides can be used in the development of functional foods including dairy products and meal replacement powders. Algal polysaccharides can help ease gastrointestinal inflammatory responses. They also assist in regulating immune function. In addition, they reduce gastric mucosal inflammation [70]. In addition, chitosan also has significant applications in the food industry. Chitosan can be used as an edible coating or preservative to cover the surface of fruits, vegetables, and meat products. It thus effectively extends the storage life of foods while cutting down on the application of chemical preservatives. This is attributed to its ability to inhibit microbial growth and alleviate oxidative stress-induced inflammatory responses [72].

In addition, carrageenan is widely used in meat products, dairy products, and foods containing gelatin. Its anti-inflammatory properties can improve food processing safety, thereby improving the water retention capacity, texture and stability of the products. Marine polysaccharides, represented by carrageenan, have become important natural alternatives to phosphates in meat product formulations. Marine polysaccharides can form a stable gel network in the meat system to absorb and lock water, and their application effect has been accurately evaluated [88]. The combination of carrageenan with plant protein or natural glycosides can further enhance its emulsifying properties. There are intermolecular interactions between proteins and glycosides in the pea protein stevioside complex. The results indicate that this effect can optimize the interfacial film-forming ability of the composite, providing a feasible modification direction for the development of high-performance marine polysaccharide-based emulsifiers [89]. Also, carrageenan can be compounded with xanthan gum to improve the elasticity of food systems, which is suitable for gummy production. Furthermore, its rheological properties in turn contribute to gel formation, maintain emulsion stability, and protect natural pigments [76].

### 4.2. Anti-Inflammatory Drugs

Inflammation is usually a beneficial biological defense reaction. However, it can also cause damage to host tissues or cause harmful reactions in specific tissues (such as transparent tissue), which leads to fever, leukocytosis and other symptoms throughout the body [90]. Research over the past few years has confirmed that marine polysaccharides have anti-inflammatory properties against inflammation in distinct regions of the human body.

#### 4.2.1. Therapeutic Agents for Gastrointestinal Inflammatory Diseases

Inflammatory bowel disease (IBD) is a common chronic non-specific inflammatory disease. The improvement in living standards and changes in dietary habits have led to a gradual increase in the incidence of IBD. This situation requires the attention of the whole society [91]. At present, the main clinical intervention for IBD is symptomatic treatment with medication, but the efficacy of the therapy is not ideal and the therapy is often accompanied by side effects [92].

Many studies have proved that marine polysaccharides can improve the disease activity index (DAI)-related clinical symptoms in mice. They can also regulate the pathological state of the colonic tissue and the permeability of the mucosa. Marine polysaccharides can also regulate many factors related to inflammatory response, such as the level of oxidative stress, inflammatory signaling pathways and gut microbial composition [93]. In addition, the therapeutic targets of algal polysaccharides include factors such as pro-inflammatory cytokines, adhesion molecules, and intestinal epithelial cells. They also include key factors such as active oxygen species and active nitrogen [94]. The anti-inflammatory effects of algal polysaccharides and their extracts can play a protective role in the stomach and intestines, and they resist the reaction of gastric juice and digestive enzymes. Unlike carrageenan, fucoidan relieves ulcerative colitis (UC) by regulating intestinal microbiota and repairing the intestinal barrier. In order to improve the biological activity of fucoidan, it was modified using the sodium hypochlorite oxidation method. By introducing aldehyde groups, they allowed fucoidan to cross-link with other substances through Schiff base reactions. This treatment can effectively improve the anti-inflammatory properties of fucoidan [71]. Hence, marine polysaccharides possess extensive application value in combating and managing gastrointestinal disorders.

In addition to being used as a drug raw material, marine polysaccharides are also widely used in the preparation of nutraceuticals with gastrointestinal inflammation-protective functions. Chitosan can regulate macrophages and intestinal microbiota. It can also inhibit the release of inflammatory factors [75]. For these reasons, it is applicable for the development of intestinal immune health products. Carrageenan has the same functional application in this field [78]. As a sulfated polysaccharide, sea cucumber polysaccharide has dual effects of anti-inflammation and immune regulation. Fucoidan from sea cucumber cooking liquid (Fuc-SC) has been developed as a high-end tonic and anti-inflammatory drug. Its efficacy has been verified in preventing gastritis caused by Helicobacter pylori SS1 (Hp SS1) infection. This efficacy highlighted its potential in gastrointestinal health maintenance. It further revealed its potential in immune support [79]. However, at this stage, the research on fucoidan from sea cucumber cooking liquid is still at the level of in vitro cell and in vivo animal experiments. The experimental results can only confirm that it has the potential to be developed into a nutritional and health-care product. For the large-scale industrial production and commercialization of sulfated polysaccharides, more targeted clinical trials must be carried out.

#### 4.2.2. Osteoarthritis Treatment Medications

In addition to being used in the preparation of the above-mentioned drugs related to gastrointestinal inflammation, marine polysaccharides are also of great value in the treatment of orthopedic inflammation. It was found that the chondroitin sulfate–chitosan–glycerol β–sodium phosphate–Fn/siHMGB1 (OCF/siHMGB1) hydrogel system not only possesses temperature-sensitive self-healing properties but also can achieve the sustained release of siHMGB1 at the joint site. This substance acts via the HMGB1/TLR4/NF-κB-p65 pathway. It promotes the M1-to-M2 polarization of macrophages. It inhibits the production of pro-inflammatory factors. It also upregulates the expression of anti-inflammatory factors. Additionally, it blocks inflammatory signaling pathways [73]. Because of these mechanisms, it is a prospective candidate for the research of arthritis therapeutic drugs. Fucoidan has important potential in the development of joint maintenance and immunomodulatory products. It inhibits the induction of MAPK, Akt and NF-κB signaling cascades through a new regulatory mechanism of the miR-22/HO-1 axis. Meanwhile, it reduces the levels of inflammatory factors in the joint microenvironment. Fucoidan effectively attenuates cartilage damage, thereby reducing the oxidative stress response of chondrocytes and synovial fibroblasts associated with osteoarthritis (OA) [6]. However, research on the use of marine polysaccharides for the treatment of osteoarthritis is still limited to laboratory-based in vitro and animal experiments, lacking evidence to support its clinical application. The optimal dose and treatment cycle of marine polysaccharides for clinical application are not yet clear [95]. Their anti-inflammatory properties even decline at high doses [96] and the safety of long-term medication also needs further verification.

#### 4.2.3. Dental Anti-Inflammatory Therapeutics

In the field of dental implants, as bacterial resistance increases, an alternative material is urgently needed. The polysaccharides with anti-inflammatory and anti-bacterial properties perfectly meet the market demand. Marine polysaccharides such as chitosan, alginate, and carrageenan can effectively resist pathogenic bacteria. In addition, they reduce the growth of pathogens through their biofilms on the surface of implants. Therefore, these polysaccharides are ideal for making functional implant coatings. These coatings have the characteristics of a rapid antibacterial effect, good anti-inflammatory effect, low toxicity and so on [74]. At present, plant polysaccharides have been released into the market as dental anti-inflammatory agents, but the development of marine polysaccharides in this field is still in its infancy. Compared with plant polysaccharides, the unique sulfate group of marine polysaccharides can endow them with stronger antibacterial and anti-inflammatory effects [97]. For example, agar, carrageenan, and alginate, which are all polysaccharides derived from brown algae and other marine sources, can replace traditional synthetic polymers. Because they are biodegradable, non-toxic and antibacterial, they can be used in the fields of dental antibacterial therapy and tissue regeneration and are environmentally friendly materials. At this stage, the application of marine polysaccharides in the dental field still faces many challenges. For instance, large-scale production is difficult. At the same time, the composition of marine polysaccharides from different sources is different. The mechanical strength of marine polysaccharide materials is not enough. These problems need to be gradually solved by modifying and strengthening such materials and developing low-cost extraction processes [98]. In addition, the optimal concentration and application method of marine polysaccharide coatings for dental implants have not been determined. This may be a key direction for future research.

### 4.3. Wound Repair and Tissue Regeneration Materials

Marine polysaccharides have excellent biocompatibility and good water retention properties. After rational modification, their antibacterial and anti-inflammatory activities have been significantly improved. Because marine polysaccharides can create the best moist environment for wound healing and can also efficiently absorb tissue exudates, they are ideal matrices for wound repair hydrogels. They play a key role in treating acute wounds such as burns and scalds and chronic wounds such as diabetic ulcers and pressure ulcers [99]. Among them, agarose, sodium alginate, chitosan and carrageenan can prepare hydrogels to promote wound repair and tissue regeneration through molecular modification or combination with other substances.

Agarose is a linear neutral polysaccharide derived from red algae. It is commonly used in the preparation of gels, with broad application prospects in the pharmaceutical, food and drink, beauty products and agricultural industries [81]. This hydrogel has excellent skin adhesion and can not only maintain a moist repair environment for the wound surface but also effectively extend the use cycle of the dressing. The hydrogel has excellent resilience, thermal stability and mechanical properties. On the one hand, in vitro experiments confirmed its good antibacterial activity and excellent biocompatibility. On the other hand, in vivo studies further verified that the hydrogel can effectively reduce the inflammatory response at the wound site and promote the wound healing process [82]. In summary, this highly stable GA-AG double-network hydrogel shows broad application prospects in the field of wound repair, suggesting that it deserves further exploration in future research.

Sodium alginate is a negative linear polysaccharide extracted from brown algae and can also be used to prepare hydrogels with similar functions as mentioned above. A physically cross-linked double-network hydrogel composed of SA (sodium alginate), CS (chitosan) and Zn^2+^ was successfully prepared by combining the semi-dissolved acidified sol–gel conversion method with the internal cross-linking method. The results showed that the hydrogel showed excellent performance in promoting wound healing and skin regeneration. Therefore, it deserves further research in the medical field as a natural biomaterial with antibacterial and anti-inflammatory activities [83]. A new hydrogel based on carboxymethyl chitosan/oxidized sodium alginate/polygallic acid/ferric ions (CMC/OSA/pGA/Fe^3+^, COGFe). They built the hydrogel into a dynamic network structure through Schiff base reactions and catechol-Fe^3+^ chelation. This structure gives COGFe good toughness, conductivity, adhesive properties and self-healing capabilities. This material can adapt to the deformation of the organism’s skin and promote the proliferation and migration of fibroblasts. Furthermore, it exhibits good biocompatibility and hemostatic activity. COGFe can regulate macrophage polarization toward the M2 phenotype. This improves the immune microenvironment, thereby exerting anti-inflammatory and antibacterial effects. At the same time, it accelerates collagen deposition and angiogenesis, shortening the wound repair cycle [84]. This research provides new ideas and strategies for the clinical treatment of infected wounds.

### 4.4. Drug Delivery System

**Figure 3:**
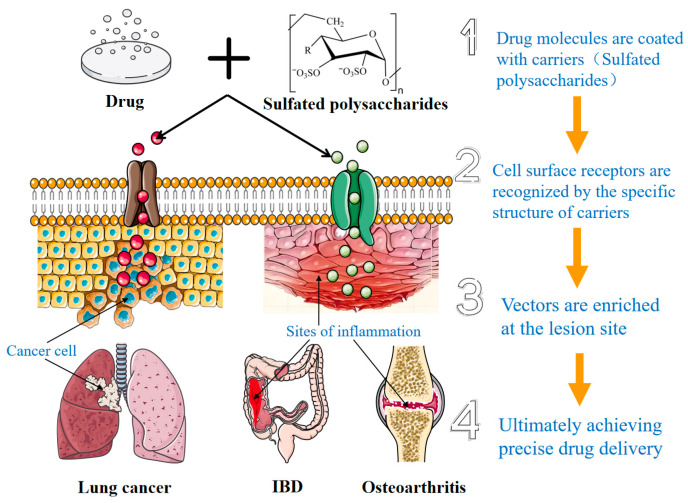
Schematic of marine polysaccharide drug precision delivery system (The red and green spheres both represent drug molecules coated with sulfated polysaccharides).

Precision drug delivery systems use biomolecules as carrier materials and are designed to deliver drugs directly to diseased sites. Such systems can significantly improve therapeutic efficacy and reduce side effects caused by direct administration. Previous studies have confirmed that marine polysaccharides have the ability to deliver precise drugs, activate immune cells and regulate immune responses, while helping to maintain the stability and activity of the loaded drugs. Marine polysaccharides have excellent biocompatibility and are easy to modify and can also be combined with gene therapy. Although structural modification is often used to optimize marine polysaccharides for use as delivery carriers, it is not a prerequisite. Many marine polysaccharides, especially sulfated polysaccharides, have many active groups that can be used to prepare composite carriers without structural modification. In addition, various other delivery systems such as microcapsules, nanoparticles, and related structures can be utilized to demonstrate the diversity of available carrier systems [100]. After structural modification, polysaccharides can be prepared into delivery carriers such as hydrogels and microspheres. These preparations further enhance the therapeutic effect on targeted diseases. In vitro experiments showed that nanoparticles based on marine polysaccharides have strong targeting ability and exhibit high cellular uptake efficiency [101]. These nanoparticles improve drug utilization and delivery accuracy. They effectively lower the toxicity of therapeutic agents. A variety of marine polysaccharides can serve as key materials in drug delivery systems, including chitosan, carrageenan, fucoidan, and laminarin. The specific mechanism of the system is shown in Figure 3.

Self-assembly and ion cross-linking are common methods for preparing alginate-based nanoparticles derived from brown algae. The layer-by-layer self-assembly method involves obtaining encapsulated nanoparticles by alternately titrating drops of polyelectrolyte polymers such as chitosan and alginate. As negatively charged polymers, alginates can interact with positively charged polymer cations, typically calcium (Ca^2+^), to form cross-links [102]. Other methods such as emulsification, spray drying and electrospinning are also widely used in this field. These nanoparticles can efficiently encapsulate drugs and improve drug delivery efficiency [103]. It was found that algae fucoidan nanoparticles combined with piperine (PL) can improve the water solubility and bioavailability of PL. This complex achieves sustained drug release, thereby enhancing the efficiency of drug uptake within cells [104]. In the treatment of inflammatory bowel disease (IBD), oral drugs must be specifically delivered to the colon to be effective. Research has shown that the specific molecular structure of sulfated polysaccharides can recognize cell surface receptors (including specific receptors, scavenger receptors, and enzyme receptors) and regulate effector molecules in cell signaling pathways [5]. Therefore, marine polysaccharides are important carrier molecules in the precision delivery system of IBD drugs. Mannose-modified trimethylchitosan (MTC) was linked with toll-expressing plasmids via ion cross-linking to construct the MTC–Tollip nanoparticle system. The experimental results showed that the system can selectively target drugs to mouse intestinal macrophages, reducing the upregulation of TLR pathway-related target genes in M1 macrophages, that is, by regulating the polarization direction of macrophages to promote the repair and reconstruction of mucosal barriers [105]. In addition, laminarin can effectively resist degradation in the gastric acid environment. Nanoparticles using it as a carrier can target inflammatory intestines through macrophage Dectin-1 receptors, thereby protecting miRNA-223 from the action of ribonucleases [106].

Fucoidan is widely used in the field of nanomedicine, as both a therapeutic agent and drug carrier. An oral microalgae hydrogel system based on fucoidan and chlorella, named Eug-Col@Fucar, for the treatment of gouty arthritis. This system promotes anti-inflammatory M2-like cells and inhibits the NLRP3-IL-1β pathway by clearing reactive oxygen species and reshaping the inflammatory microenvironment. This effect can significantly reduce the release of pro-inflammatory factors, thereby inhibiting the pathological progression of gouty arthritis. Moreover, the hydrogel can also synergistically reduce uric acid levels, achieve precise control and release of drugs, and prolong the intestinal retention time of therapeutic agents. If this study is applied clinically, it can alleviate the side effects of oral colchicine, thereby reducing the damage caused by the drug in the enterohepatic circulation [107]. However, the pathological mechanism of osteoarthritis is intricate, involving multiple processes such as inflammatory response and cartilage degeneration. In addition, current research is still focused on the fundamental level, and there are related issues in biosafety. Therefore, the design and practical application of marine polysaccharide nanosystems still face many challenges [108].

Combining marine polysaccharide drug delivery systems with immunotherapy enables the two to exhibit a potential synergistic effect. Marine polysaccharides themselves have inherent immune regulatory effects, and the combination of two therapies can enhance the recognition ability of the immune system and the ability to accurately attack tumors. In adoptive cell therapy (ACT), such systems can effectively package immune effector cells (T cells and NK cells) and then deliver them to nanoparticles or hydrogels. This type of carrier has good biocompatibility, effectively improving cell survival rate and tumor targeting efficiency [109]. For example, polysaccharides isolated from the marine fungus *Phoma herbarum* YS4108 can promote dendritic cell maturation. They also enhance the antigen-presenting capacity of these cells. Therefore, a drug delivery system based on this polysaccharide can promote the activation and proliferation of T cells. In turn, they enhance anti-tumor and anti-inflammatory effects [101]. Dietary L-fucose can enhance the immunostimulatory activity of dendritic cells. It also strengthens their ability to activate T cells [110]. Multiple studies have confirmed that chitosan nanoparticles can be used as effective carriers to accurately deliver Hif-1α siRNA to cancer cells. Chitosan/HIF-1α siRNA nanocomplexes were successfully prepared using an siRNA adsorption method. This method achieves efficient loading of siRNA on chitosan nanoparticles. When combined with a chemotherapeutic agent that induces immunogenic cell death (Ptx) and a TLR7 ligand (Imq), the nanocomplex demonstrated significant efficacy. It was able to effectively overcome the tumor microenvironment, which is the major obstacle in cancer treatment [111].

## 5. Challenges and Future Prospects

Marine polysaccharides have received extensive research attention due to their excellent anti-inflammatory activity. This review focuses on the structural characteristics of marine polysaccharides, as well as their anti-inflammatory mechanisms and practical applications. In general, this overview provides a deep understanding of marine polysaccharides, which can facilitate their applications in anti-inflammatory drugs and other areas. However, there are still many challenges.

### 5.1. Extraction and Standardization

In the extraction and purification process, it is difficult to maintain the high purity and structural uniformity of marine polysaccharides. That is to say, the efficient separation process of marine polysaccharides faces difficulties. This may not only hinder the advancement of subsequent related research but also affect the stability of processed products. Currently, marine polysaccharides are mainly extracted by water extraction and alcohol precipitation, followed by separation and purification by ion exchange chromatography. The complexity of the entire operation process is a direct reason for the low yield of polysaccharides [112]. Furthermore, to accurately analyze the structure of marine polysaccharides, it is often necessary to combine multiple chromatographic techniques. Such analysis methods are complex and time-consuming [113]. Therefore, there is an urgent need to integrate artificial intelligence technology into the separation and purification processes of marine polysaccharides, such as predictive modeling of process parameters, dynamic adjustment of operational conditions in real time, and rapid, data-driven selection of optimal purification strategies. At the same time, the methods for analyzing polysaccharide structures should be improved to achieve full-process optimization.

### 5.2. The Potential of Marine Polysaccharide Synthesis Genomics

In recent years, genome sequencing and gene mining technologies have made breakthroughs. This provides important technical support for researchers to identify key genes for polysaccharide synthesis and analyze metabolic pathways. The genome of the chitin-degrading strain KSP-S5-2 was analyzed, and the genetic plasticity and gene library characteristics that support the evolution of polysaccharide degradation in the Cytovibrionaceae family were discovered. This study confirmed not only the genomic potential of this strain but also the chitin degradation mechanism. At the same time, the diversity of polysaccharide-degrading families within the strain was demonstrated [114]. Although there are many sulfated and phosphorylated polysaccharides in nature, in practical applications, technical problems such as low yield and scarce raw materials are often encountered. These difficulties also restrict the further application of this type of polysaccharide [115]. Therefore, systematic analysis of polysaccharide synthesis pathways and key genes is of far-reaching significance for promoting polysaccharide production and targeted transformation.

Genomic methods can facilitate researchers in discovering new biosynthetic gene clusters (BGCs) and using metabolic engineering to increase the production of target polysaccharides, addressing many key limitations [116]. For example, comparative genomics can identify conserved BGCs responsible for assembling complex polysaccharide backbones, while functional genomics can characterize the catalytic role of key biosynthetic enzymes. In addition, synthetic biology strategies, such as heterologous expression of these BGCs in robust microbial hosts, have the potential to overcome natural production constraints and achieve scalable and sustainable production of high-value marine polysaccharides [21]. These genomic tools not only deepen our understanding of polysaccharide biosynthesis but also open up new avenues for rational design and optimization for industrial production, which is beneficial for addressing the core challenges of low yield and scarce raw materials [117].

### 5.3. Clinical Trials

To date, most studies have been limited to in vitro experiments or animal models for validating the diverse functions and potentials of marine polysaccharides. Further clinical trials are required to validate their efficacy and safety. Furthermore, researchers have yet to fully understand the molecular mechanisms underlying the interactions between polysaccharides and related cells [118]. In addition, the anti-inflammatory properties of marine polysaccharides vary significantly among individuals. For example, inter-individual differences in the human gut microbiota lead to different metabolic effects of dietary soluble polysaccharides in the intestine [119]. Future research ought to give precedence to personalized translational clinical research to connect preclinical findings with real-world human applications. In-depth studies on the structure–activity relationships and receptor-level mechanisms of polysaccharide–cell interactions will be essential to unlock their full therapeutic potential and guide the rational design of next-generation marine-derived products [120].

### 5.4. Food Industry Applications

In the food industry, the anti-inflammatory property of marine polysaccharides is not only of great help during processing. At the same time, this property is also one of the main functions of products processed by the food industry. However, there are still many challenges in terms of its own characteristics and production and processing.

Firstly, the production of certain marine polysaccharides (such as fucoidan and carrageenan) relies on specific algae or marine organisms, which are subject to seasonal variations, geographical constraints, and limitations in harvesting or cultivation conditions, leading to unstable raw material supply [121]. Moreover, some marine polysaccharides may carry marine pollutants such as heavy metals or algal toxins or trigger allergic reactions (e.g., chitin derivatives). The residual by-products produced by chemically modified marine polysaccharides also have corresponding hazards and require strict monitoring [122]. Furthermore, at this stage, some marine polysaccharides have not yet obtained official certification qualifications as food additives or new food ingredients. Laboratory-level extraction and modification technologies cannot meet the actual needs of industrial production in terms of production capacity and stability. Additionally, continuous support facilities and low-cost integrated equipment systems are not yet complete [123]. Future exploration can use genetic engineering technology to transform microorganisms (such as E. coli or yeast) to achieve heterologous and efficient synthesis of high-value marine polysaccharides [124]. This approach would reduce dependence on natural resources, accelerate safety assessments and international standard recognition, and expand their applications in functional foods, meal replacements, and other related fields. Opportunities always coexist with challenges. Marine polysaccharides, as effective anti-inflammatory components, have infinite possibilities for development in the modern food industry.

## Figures and Tables

**Table 1:** Structural characteristics of main types of marine polysaccharides.

Source	Type	Structural Information	References
Algal polysaccharides	Alginate	A linear polymer formed by the alternating linkage of β-D-mannuronic acid (M) and α-L-guluronic acid (G) through 1,4-glycosidic bonds	[13]
	Fucoidan	A sulfated polysaccharide formed by connecting L-fucose as the main structural unit through 1,3- or 1,4-glycosidic bonds. The molecule also contains a small amount of residues such as galactose, mannose, and glucuronic acid	[14]
	Laminarin	A type of β-1,3-glucan, composed of D-glucose linked together via 1,3- and 1,6-glycosidic bonds	[15]
	Carrageenan	A linear sulfated polysaccharide formed by the alternating linkage of β-D-galactopyranose (namely G-units) and α-D-galactopyranose (namely D-units) through 1,3- and 1,4-glycosidic bonds, or formed by 3,6-anhydro-α-D-galactopyranose (namely DA-units). It can be divided into three main types (κ-type, ι-type, and λ-type)	[14,16]
	Agarose	A linear polysaccharide without sulfate groups, formed by the connection of β-D-galactose and 3,6-anhydro-α-L-galactose through 1,3- and 1,4-glycosidic bonds	[11]
	Agaropectin	Different from agarose, it contains a small amount of sulfate groups and carboxyl groups	[11]
	Ulvan	It is mainly a polyanionic heteropolysaccharide composed of rhamnose, glucuronic acid, iduronic acid, and xylose and contains sulfate groups, and its skeleton is mainly composed of α- and β-(1,4)-bonds, forming repeated disaccharide units; the main repeating disaccharide units are divided into ulvanobiuronic acid 3-sulfate type A (A_3s_), and type B (B_3s_), and in some cases type U (U_3s_ and U_2′s,3s_)	[17,18]
Animal polysaccharides	Chondroitin sulfate	A linear polysaccharide: the main chain is composed of two monomers, D-glucuronic acid and N-acetyl-D-galactosamine, which are alternately linked by β-1,3- and β-1,4-glycosidic bonds. Most of the sulfate groups on its molecule are attached to the C4 or C6 position of N-acetyl-D-galactosamine	[12]
	Dermatan sulfate	The main chain is composed of L-iduronic acid (or D-glucuronic acid) and N-acetyl-D-galactosamine, linked by β-1,3- and β-1,4-glycosidic bonds. Its sulfate groups are mainly attached to the C4 position of N-acetyl-D-galactosamine, and sulfation also occurs at the C2 position of some iduronic acid molecules	[19]
	Chitosan	A linear polysaccharide, specifically known as (1,4)-2-amino-2-deoxy-β-D-glucan	[20]
Microbial polysaccharides	*Vibrio* sp. KMM 8419	The sulfate group is attached to the C-3 of the L-rhamnose residue, and the C-4 of L-rhamnose has an acetylation modification	[21]

**Table 2:** Structure and functional impact of partial marine polysaccharides.

Polysaccharide	Structural Parameters	Functional Impact
Alginate	High-molecular-weight	Gel properties and viscosity
	Low-molecular-weight	Solubility and the biological activity is more easily exerted
Laminarin	Long-chain and high-molecular-weight	Exerts both immune-enhancing and anti-inflammatory activities
Chitosan	High-molecular-weight	Poor solubility but excellent film-forming and flocculating properties
	Low-molecular-weight	Water solubility and easier penetration through bacterial cell walls
Fucoidan	L-fucose as its main constituent unit and small amounts of miscellaneous sugar components	Increases the interaction sites between the polysaccharide and inflammatory factors
Carrageenan	λ-type	Water solubility; strong electrostatic interactions; anti-inflammatory applications
Agaropectin	Sulfate groups; carboxyl groups	Interferes with the formation of the double-helix structure, reducing the gel strength; enhances the adhesion ability between agaropectin cells
Chondroitin sulfate	Flexible random coil in aqueous solution; different substitution position of the sulfate groups	Regulates dynamic conformational changes precisely
Ulvan	Different glycosidic bond connection modes and sulfation substitution	Recognizes and binds to inflammatory mediators and immune cells; anti-inflammatory activity

**Table 3:** Anti-inflammatory activity and application of marine polysaccharides and their derivatives.

Types of Marine Polysaccharides and Their Derivatives	Anti-Inflammatory Activity	Main Application Areas	References
Fucoidan	Stabilizing IκB-α can inhibit aspirin-induced NF-κB activation and reduce gastric mucosal inflammation. The MAPK, Akt and NF-κB pathways are inhibited by regulating the miR-22/HO-1 axis to alleviate cartilage damage. It also inhibits colonic inflammation, regulates microflora and repairs the intestinal barrier, relieving ulcerative colitis.	Food industry (dairy products, meal replacement powders and other functional food ingredients); health-care products (joint maintenance and immunomodulation products); biomedical (inflammatory bowel disease therapeutics)	[6,70,71]
Chitosan	Low-molecular-weight chitosan inhibits microbial growth and reduces oxidative stress and inflammation. It regulates macrophages and intestinal microbiota, transforms M1 to M2 macrophages, inhibits pro-inflammatory factors, enhances anti-inflammatory factors, and promotes Treg proliferation and immunosuppressive function.	Food industry (edible coatings and preservatives); health products (intestinal immunity health products); biomedical (arthritis drugs, functional coatings for dental implants, wound repair materials, and drug delivery systems)	[72,73,74,75]
Carrageenan	Its anti-inflammation can improve food processing safety; λ-carrageenan oligosaccharide reduces IL-6 and TNF-α by inhibiting the MMP-9 and NF-κB/MAPK pathways and improves psoriatic dermatitis in mice.	Food industry (meat products, dairy products, foods containing gelatin, gummies, beverages, etc., which play a role in thickening, stabilizing, gel formation, etc.); health products (skin inflammation-related health products)	[76,77,78]
Fucoidan from sea cucumber cooking liquid	Prevents gastritis caused by Helicobacter pylori SS1 (Hp SS1) infection and inhibits inflammatory responses.	Health products (high-end tonics and health supplements)	[79,80]
Chondroitin sulfate oxide	The HMGB1/TLR4/NF-κB-p65 pathway regulates macrophage transformation and blocks inflammatory signaling.	Biomedical (arthritis drug)	[73]
Agarose	Good biocompatibility; can form hydrogels with anti-inflammatory and antibacterial properties, reducing inflammation and accelerating wound healing.	Biomedical (wound repair and wound healing materials)	[81,82]
Sodium Alginate	Forms hydrogels with antibacterial and anti-inflammatory properties that promote wound repair and skin regeneration, while also regulating the polarization of macrophages towards the M2 phenotype to improve the immune microenvironment.	Biomedical (wound repair materials)	[83,84]
Ulvan	It can inhibit nociception caused by acetic acid/formalin, block the bradykinin pathway, inhibit foot edema caused by dextran, inhibit the release of TNF-α and IFN-γ induced by LPS, upregulate the anti-inflammatory factors TGF-β1 and IL-10, reduce MMP-3 and IL-6 levels, increase chondrocyte COL2A1 levels, reduce TG/TC/LDL-C in OA rats, inhibit pro-inflammatory cytokines/NO/MMP-3/CTX-II, and reduce cartilage proteoglycan loss.	Biomedical (osteoarthritis therapeutic drugs, second-degree burn wound repair gel and electrospun nanofiber wound dressing materials); health products (blood lipid regulation, joint health, and analgesic and anti-inflammatory health products)	[85,86,87]

## Data Availability

This study did not generate any new datasets. All data analyzed are from publicly available sources, as cited in the manuscript.
